# Participatory Research in Clinical Studies: An Innovative Approach to Co‐creating Nutritional and Physical Activity Recommendations for Older Adults With Sarcopenia (FOOP‐Sarc Project)

**DOI:** 10.1111/hex.70187

**Published:** 2025-04-04

**Authors:** Maria Besora‐Moreno, Cristina Sepúlveda, Judit Queral, Claudia Jiménez‐ten Hoevel, Anna Pedret, Lucia Tarro, Rosa M. Valls, Rosa Solà, Elisabet Llauradó

**Affiliations:** ^1^ Universitat Rovira i Virgili, Facultat de Medicina i Ciències de la Salut, Functional Nutrition, Oxidation, and Cardiovascular Diseases Group (NFOC‐Salut) Reus Spain; ^2^ Institut Investigació Sanitària Pere i Virgili (ISPV) Reus Spain; ^3^ Hospital Universitari Sant Joan de Reus Reus Spain

**Keywords:** co‐creation, nutrition, participatory research, physical activity, sarcopenia

## Abstract

**Background:**

Participating in co‐creation processes can improve the knowledge, satisfaction and healthcare outcomes of volunteers. However, this methodology is still underused in nutritional clinical studies.

**Objective:**

This study aimed to use participatory research as an innovative approach to co‐creating nutritional and physical activity (PA) recommendations for the FOOP‐Sarc project and to assess their usability and volunteers' satisfaction and engagement experience (SEE) during the co‐creation process.

**Design:**

The co‐creation process was based on four stages: (s1) co‐ideation, (s2) co‐design, (s3) co‐implementation and (s4) co‐evaluation (Ref.: NCT05485402).

**Setting and Participants:**

Thirteen volunteers with sarcopenia were included (stages 1–2 [*n* = 7], stage 3 [*n* = 3 intervention, *n* = 3 control] and stage 4 [*n* = 13]).

**Measures:**

The co‐ideation (s1) and co‐design (s2) stages focused on designing recommendations adapted to the volunteers' preferences; the co‐implementation (s3) stage included the implementation and comparison of the co‐created or standard recommendations for 3 weeks to test the recommendations' acceptance; and the co‐evaluation (s4) stage focused on usability, SEE, and adherence.

**Results:**

The volunteers co‐created recommendations for improving sarcopenia according to the barriers identified related to diet and PA. The recommendations' usability and the SEE of volunteers were high in all cases.

**Conclusions:**

The participatory research approach used in this nutritional intervention study, demonstrates a high usability of the co‐created recommendations for sarcopenia and high SEE of the volunteers, particularly in the volunteers who participated in co‐ideation (s1) and co‐design (s2), the most key stages of the co‐creation process.

**Patient or Public Contribution:**

The volunteers in this study participated in the co‐creation of nutritional and PA recommendations to improve sarcopenia, which they must subsequently follow.

**Trial Registration:**

ClinicalTrials.gov identifier: NCT05485402.

## 1 Introduction

In recent years, public participation in treatment and screening decision processes has increased [1]. Participatory research refers to the collaboration of researchers with people affected by the reason for study (patients, community members or health professionals), and other people interested in the subject, who are called stakeholders [2]. Furthermore, in 2020, the European Commission created the Science with and for Society (SwafS) programme to align science with the needs of society as a means of making science more attractive [3]. This can ensure that the people who participate as decision aids are better informed, possess improved knowledge and have better risk perceptions [1]. Additionally, this approach can decrease decisional conflict between practitioners and older people [4, 5, 6], increase patient satisfaction [7, 8] and improve their healthcare outcomes [9].

Various methods are used to increase public participation in decisions, such as co‐creation, which is defined as a collaborative problem‐solving process involving researchers, participants and stakeholders, progressing from problem identification and solution proposal to solution implementation and evaluation [10, 11]. The co‐creation process comprises four collaborative stages: co‐ideation (the generation of the idea), co‐design (the design of the programme or intervention and its methodology), co‐implementation (the implementation of the intervention or programme following the methods previously designed) and co‐evaluation (the final analysis and the interpretation of data to assess the co‐creation process) [10].

The inclusion of co‐creation in research projects, clinical studies or educational health interventions can increase their quality [12], rigour and relevance [13], as well as adherence to interventions [8, 9], and should form a key aspect of future research [14]. Improving volunteers' adherence to nutritional clinical studies is a priority; enroling volunteers, enhancing their engagement and ensuring their satisfaction ensures increased adherence and retention during clinical trials [15, 16]. However, limited number of studies have used co‐creation as a tool to design health recommendations to be implemented in intervention studies. Therefore, the present study aims to improve this scarcity through co‐creation generating new knowledge of the subject.

According to the revised consensus of the European Working Group on Sarcopenia in Older People (EWGSOP2), published in 2019, sarcopenia is a multifactorial, progressive and generalized musculoskeletal disorder characterized by low muscle strength, low muscle quantity or quality, and/or low physical performance due to aging [17]. The present study is part of a research project entitled “Foods such as virgin olive oil rich in phenolic compounds, and prebiotic supplementation: a dietary strategy to tackle Sarcopenia in older adults” (FOOP‐Sarc). The FOOP‐Sarc project is a 24‐week (12 weeks of intervention and 12 weeks of follow‐up after intervention cessation) randomized, controlled, parallel and double‐blind intervention study based on the supplementation of virgin olive oil rich in phenolic compounds alone or in combination with a prebiotic (fructooligosaccharides and inulin) and nutritional and physical activity (PA) recommendations in older (aged 60–80) sarcopenic subjects.

The main objective of the present study is to use participatory research as an innovative approach to co‐creating nutritional and PA recommendations for the FOOP‐Sarc project and to assess their usability and the volunteers' satisfaction and engagement experience (SEE) with the co‐creation process.

## 2 Materials and Methods

### 2.1 Study Design

The present study involved the co‐creation of nutritional and PA recommendations to improve sarcopenia parameters in the FOOP‐Sarc project volunteers with sarcopenia. Thus, the co‐creation process was carried out by a subsample of volunteers participating in the FOOP‐Sarc project. Nutritionists on the research team participated as facilitator agents guiding the volunteers during the co‐creation process, especially in the co‐ideation and co‐design stages. The present study was conducted in accordance with the Helsinki Declaration and Good Clinical Practice Guidelines of the International Conference of Harmonization (GCP ICH). This study was approved by the Ethics Committee for Research with Medicines (Comité de Ética de Investigación con medicamentos [CEIm]) of the Institut d'Investigació Sanitària Pere Virgili (IISPV) (Ref. 033/2022). The study protocol was registered on ClinicalTrials.gov (NCT05485402). All volunteers signed their informed consent before participation, and all of the authors declared that they had no conflicts of interest. The present intervention study was defined using the PRoblem, Objective, Design, (end‐)Users, Co‐creators, Evaluation and Scalability (PRODUCES) criteria [18] (Table 1).

**Table 1:** PRODUCES criteria.

PRoblem	Sarcopenia in older adults
Objective	Design nutritional and physical activity recommendations for older adults with sarcopenia
Design	Participatory action research (co‐creation methodology)
(End‐) Users	Older adults (60–80 years) with sarcopenia
Co‐creators	Older adults' volunteers with sarcopenia and dietitians and nutritionists of the research team as facilitators
Evaluation	Individual assessment of the recommendations usability and the volunteers' satisfaction and engagement experience in a co‐creation process. Also, the assessment of the adherence to recommendations.
Scalability	Generalizable model

### 2.2 Eligibility Criteria

The inclusion and exclusion criteria were the same as in the FOOP‐Sarc project because the participants were a subsample of volunteers from that project. The volunteers were included according to the following criteria: (a) men and women aged ≥ 60 years and ≤ 80 years who; (b) provided written informed consent before the initial visit and (c) met at least one of the sarcopenia assessment criteria (low muscle strength based on grip dynamometry [men < 30 kg; women < 20 kg] and/or low skeletal muscle mass index [SMI] based on bioimpedance analysis [BIA] [men < 8.87 kg/m^2^; women < 6.42 kg/m^2^] and/or low physical function based on gait speed [≤ 0.8 m/s]) [19]. Volunteers who did not meet all of the inclusion criteria were excluded. The sarcopenia cut‐off points used were from the previous 2010 consensus on sarcopenia (EWGSOP1) [19].

### 2.3 Study Procedure

The present study included the four key stages of a co‐creation process: co‐ideation, co‐design, co‐implementation and co‐evaluation. The co‐creation methodology was selected because it is a flexible and adaptative methodology that involves users from the beginning of the process, allowing the developed solutions to respond to the needs identified and to be validated throughout the co‐creation process [20]. The co‐creation process lasted for 6 weeks and involved a maximum of 5 visits: a screening visit (V0) and one visit for each co‐creation stage (co‐ideation [V1], co‐design [V2], co‐implementation [V3] and co‐evaluation [V4]). The volunteers who participated in the co‐ideation and co‐design stages had a total of 4 visits (V0, V1, V2 and the co‐evaluation [V4] at the end of the co‐design stage). The volunteers who participated in the co‐implementation stage had a total of 3 visits (V0, V3 and V4). All the co‐creation process visits were carried out at the Centre MQ of Reus medical centre.

The recommendations to be co‐created were based on the nutritional and PA needs of subjects with sarcopenia. In the context of the FOOP‐Sarc project, the NFOC‐diet, defined by the nutritionists on the research team according to the nutritional needs of the subjects, was based on the recommendations of the Dietary Approaches to Stop Hypertension (DASH) diet [21] and the incorporation of foods rich in protein (in particular, leucine [22]), vitamin D, polyunsaturated fatty acids, phosphorus and iron, all micronutrients that favour muscle mass and its functionality [22, 23, 24]. High‐fat foods were not excluded from the DASH diet. The only foods excluded were those rich in saturated fat such as viscera. Table 2 includes the list of NFOC‐diet foods. In addition, PA recommendations were based on 150 min/week of moderate to vigorous PA with at least two sessions of motor strength per week to avoid a loss of muscle mass [25].

**Table 2:** List of NFOC‐diet foods.

Protein foods
Lean meat (100–150 g): turkey, chicken, rabbit, etc.
Red meat (100–150 g): pork, lamb, beef, etc.
Cold meat (30–50 g): cooked ham, cured ham, cured sausage, etc.
Eggs (1 unit): whole egg of any type
Whitefish (100–150 g): monkfish, hake, sea bass, cod, sole, gilt‐head bream, panga, halibut, turbot, etc.
Bluefish (100–150 g): anchovy, salmon, tuna, trout, sardine, herring, mackerel, etc.
Canned fish (1 unit): tuna, sardine, anchovy, salmon, etc.
Seafood (100–150 g): squid, cuttlefish, octopus, shrimp, prawn, conger, lobster, mussel, calm, etc.
Legumes (80 g raw or 160 g cooked): chickpea, lentils, white beans, soy, broad bean, lupin, etc.
Dairy and dairy products: whole/semi‐skimmed/skimmed milk (200 mL), fortified milk with vitamin D (200 mL), soy beverage (200 mL), whole plain yogurt (125 g), cheese (50–70 g).
Nuts and seeds (20–30 g): almond, hazelnut, walnuts, peanut, peanut butter, cashew, pistachio, pine nut, chia seeds, flax seeds, pumpkin seeds, sesame seeds, etc.

Cereals and tubers
Pasta and rice (whole grain if it is possible) (80 g raw or 160–200 g cooked)
Bread (whole grain if it is possible) (40–60 g) or rusk (30 g)
Quinoa (80 g raw or 160 g cooked)
Corn (70 g)
Oat flakes (30–40 g)
Potato and sweet potato (170 g)
Breakfast cereals fortified with vitamin D (30–40 g)

Vegetables and fruits
Any type of vegetables and fruits (200 g) but specifically some of them: chard (60–80 g), spinach (60–80 g), arugula (60–80 g), lamb's lettuce (60–80 g), mushrooms (140–150 g), tomato (100–150 g), avocado (150–200 g) and banana (150–200 g)

Others
Tofu or tempeh (150 g)
Cocoa (15–20 g, 2 level tablespoons or 1 full tablespoon)
Date (2–3 units)
Raisins (40 g)

To assess the usability of the recommendations, a 5‐point Likert scale questionnaire based on the validated 10‐item System Usability Scale was used [26]. In total, 8 out of 10 questions were used in the co‐ideation and co‐design stages because questions 6 and 9 could not be applied at these stages. In addition, in the co‐implementation stage, 9 of 10 questions were used because question 6 could not be applied. There is no validated questionnaire to assess SEE in a co‐creation process; therefore, a non‐validated 15‐item Engagement Experience Survey rated with a 7‐point Likert scale reported in the scientific literature was used [27]. However, 14 out of 15 questions were posed to the co‐implementation volunteers because question 8 could not be applied at this stage.

In addition, the acceptance of the recommendations was assessed at the co‐implementation stage, and an additional analysis was conducted to determine volunteers' adherence to the recommendations, which was assessed using a 50‐item non‐validated food frequency questionnaire (FFQ) related to the principal diet components of the NFOC‐diet for sarcopenic subjects and the Spanish version of the International Physical Activity Questionnaire for the Elderly (IPAQ‐E) [28]. The FFQ was created by the nutritionists of the research team using other validated FFQs [29, 30, 31, 32, 33, 34, 35, 36], with the general and specific nutrients chosen according to sarcopenia recommendations, such as protein (in particular, leucine) and vitamin D, polyunsaturated fatty acids, phosphorus and iron [23, 24]. This FFQ included different food groups such as meat, fish, eggs, dairy products, cereals, tubers, legumes, nuts, seeds, vegetables and fruits, fortified foods and others (Table S1). The mean daily intake of energy and nutrients was calculated using the 2020 CIQUAL food composition table [37]. Adherence was assessed at the beginning and the end of the co‐implementation stage.

### 2.4 Sample Size

According to the scientific literature, and to account for possible dropouts, it was recommended that a total of 10–12 subjects take part in all 4 stages of the co‐creation process [18]. It is necessary to emphasize that the sample of volunteers who co‐implemented the recommendations was different from the sample of volunteers who co‐ideated and co‐designed the recommendations to avoid cross‐contamination during co‐implementation. Thus, in the co‐evaluation stage, all volunteers from the three previous stages of the co‐creation process were included.

### 2.5 Statistical Analysis

#### 2.5.1 Qualitative Data

The qualitative data in this study refer to the interpretation of the semi‐structured interviews in the co‐ideation stage based on three different phases. First, the raw data (the exact information cited by the volunteers) were categorized according to the frequency of recurrence. Second, the descriptive information, which is a summary of the comments from the analyst who took notes during the interview, was interpreted as raw data in summary form and provided representative feedback from volunteers. Finally, the interpretive method refers to the analyst providing an interpretation that aided in understanding the interview. The information obtained was based on a combination of the transcription notes and other background information.

#### 2.5.2 Quantitative Data

Categorical variables were presented as percentages (%), and continuous variables were presented as the mean ± standard deviation (SD) for normal distribution variables, whereas non‐normal distribution variables were expressed as the mean or median ± interquartile range (IQR). For non‐normal distribution variables, the Wilcoxon test is used to assess within‐group changes in quantitative variables from the beginning to the end of the co‐implementation stage. The Mann–Whitney *U* test was also used to assess differences between groups, and the Mc‐Nemar test was used to assess between‐group changes in qualitative variables from the beginning to the end of the co‐implementation stage due to the non‐normal distribution sample. A *p*‐value of < 0.05 was considered statistically significant. All of the analyses were performed using IBM SPSS statistics software version 27.

## 3 Results

**Figure 1:**
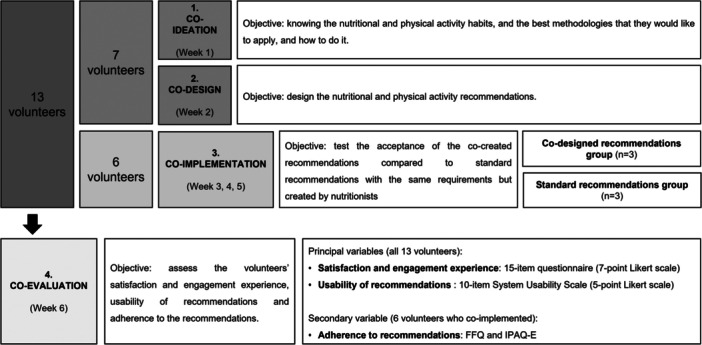
Co‐creation process.

**Figure 2:**
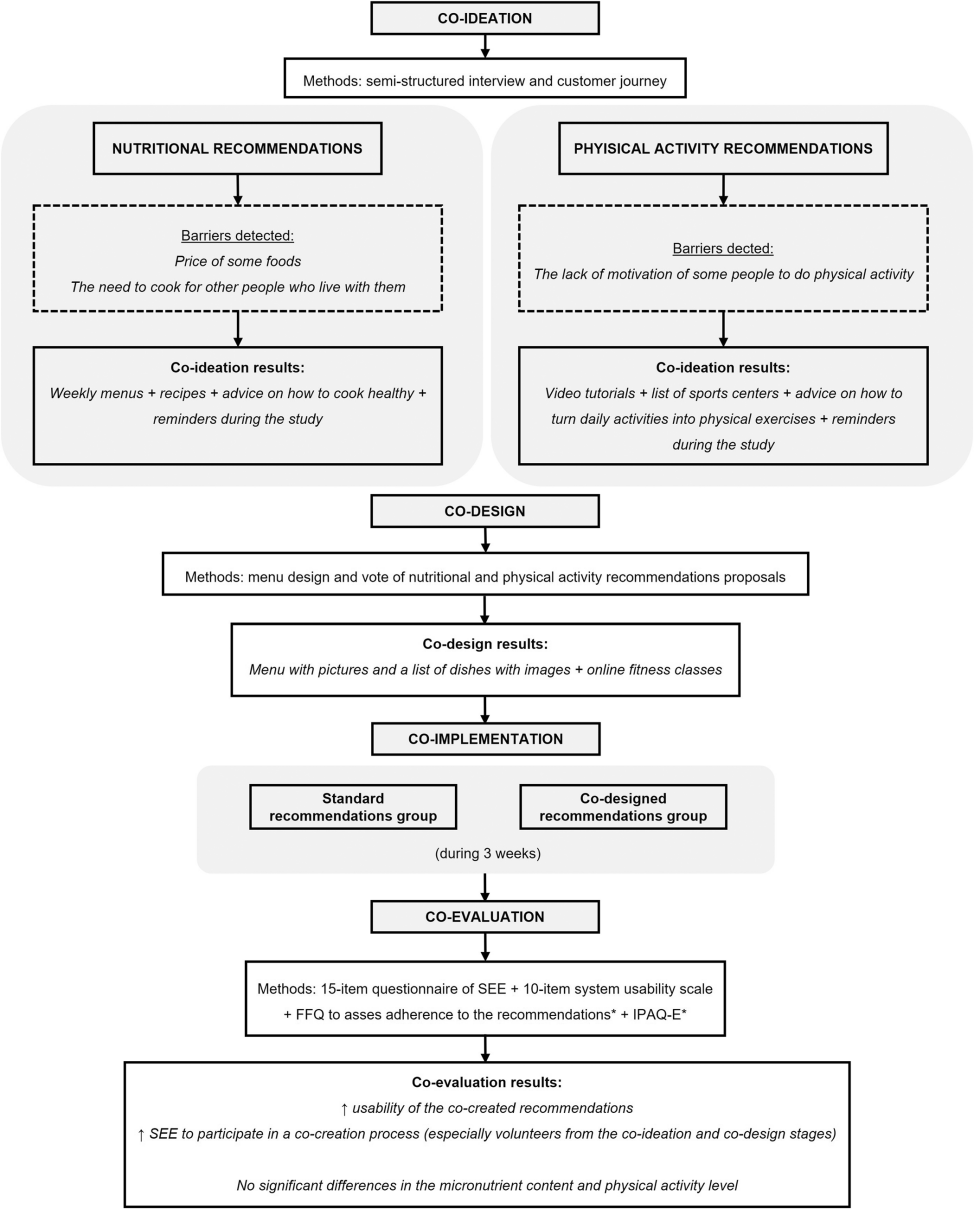
Flowchart of the results of the co‐creation process. FFQ, food frequency questionnaire; SEE, satisfaction and engagement experience; IPAQ‐E, International Physical Activity Questionnaire for the Elderly. *Only volunteers who co‐implemented.

Thirteen home‐dwelling older adults with sarcopenia aged between 60 and 80 years were included in the co‐creation process (Figure 1). In this context, seven volunteers were included in the co‐ideation and co‐design stages (one volunteer only in the co‐design stage), while six different volunteers participated in the co‐implementation stage. Finally, all 13 volunteers included were co‐evaluated. Figure 2 shows all the stages of the co‐creation process and its results.

### 3.1 Co‐ideation (Session Lasting 1 h and 40 min [V1])

The principal objectives of this session were to ascertain the frequency of food consumption, the structure of meals, the frequency of PA, the volunteers' preferences, the best methodologies that could be applied, barriers and how to improve them, and if the volunteers needed support during the study.

First, volunteers had to present themselves and define their nutritional and PA habits as briefly as possible. Second, to collect as much information as possible for the co‐design session, a semi‐structured group interview was used (Table 3). Moreover, the customer journey technique was also used in the form of an individual diary focused on the volunteer's daily habits to determine their diet and PA, used as a guide to create the recommendations [38] (Table S2). The volunteers were asked to provide their ideas using sticky notes.

**Table 3:** Topics and questions of semi‐structured interview.

General topics	Specific topics	Questions
**Food habits**	*Food frequency consumption*	– From the list of foods, we show you, which foods do you eat frequently (daily and/or weekly)? – From the list of foods, we show you, what foods do you eat occasionally (monthly)? – From the list of foods, we show you, which foods do you never or almost never eat?
	*Introduction of new food*	– Would you like to introduce foods that you do not usually use? – How would it be easier for you to introduce these foods? – At what time of the day would you like to add the foods of the list?
**Physical activity habits**	*Frequency of physical activity*	– Do you practice physical activity regularly? How often? – If you do not exercise regularly, would you be willing to introduce this new habit? How often?
	*Type of exercises*	– From the list of exercises, we show you, what exercises do you see yourself able to do?
**Barriers**	*Nutrition*	– What barriers do you find in relation to eating habits or foods that we propose? (also, think about people with more difficulties than you)
	*Physical activity*	– What barriers do you find in relation to physical activity habits or exercises that we propose? (also, think about people with more difficulties than you)
**Recommendations**	*Nutritional recommendations*	– How would you like to receive information regarding dietary recommendations?
	*Physical activity recommendations*	– How many days a week could you spend doing physical activity? – How would you like to receive information on physical activity recommendations?
**Follow‐up**	*Reminders*	– Would you like to receive reminders so you don't forget to follow the recommendations?

Regarding meal distributions, according to the semi‐structured interview and the customer journey technique, most volunteers ate four meals/day, only one volunteer ate five meals/day and another one ate three meals/day. If they missed a meal, it was usually the mid‐morning snack or the afternoon snack. During weekends, the volunteers reduced the number of meals they consumed. With regard to nutritional habits, all six volunteers had a varied diet and included different food groups. However, only five volunteers ate dairy and dairy products, only four volunteers ate nuts and seeds, and only two volunteers ate fortified foods. Also, some foods (such as soybeans, tempeh, tofu and soy drinks) were never or hardly ever consumed by the volunteers, but some of them were interested in including tofu and soybeans in their diets. Additionally, three volunteers did not eat wholegrain cereals.

To introduce new foods, the volunteers thought that mixing these foods with others that they usually ate could be a solution. Another suggestion was to have a menu available for all the members of the house. Regarding the best time to add new foods, three volunteers preferred during lunch, two volunteers during breakfast and one during dinner.

Focusing on PA habits, all the volunteers practiced PA regularly during the week for between 1 and 3 h a day alone or with friends. The most frequently practiced exercises were flexibility exercises, exercises with weights, dancing, swimming and stretching, either in local sports or social centres or alone in the house. Five volunteers also walked every day. If the volunteers were not practicing these exercises at this point, they were motivated to introduce them. Regarding sedentary time, the volunteers spent an average of 4.5 h sitting every day, either alone or with a family member. Additionally, during this time, five volunteers read and watched TV during the afternoon and night, two volunteers used the computer during the afternoon and two volunteers used their mobile frequently during the day.

The main barriers to following the recommendations reported by the volunteers were the price of some foods, the need to cook for other people living with them, and a lack of motivation to engage in PA.

Finally, the volunteers concluded which methodologies would be most effective to ensure that the nutritional and PA recommendations were followed up. In particular, the volunteers wanted to receive nutritional recommendations with some examples of weekly menus with recipes and advice on how to eat healthily; they also decided that the best way to follow the PA recommendations was by using video tutorials about how to perform the exercises, a list of sports centres in the city, and advice on how to turn daily activities into physical exercises. Additionally, they suggested that it could be useful to receive reminders during the study to motivate them to follow the nutritional and PA recommendations.

All ideas generated during the co‐ideation stage were transcribed, analysed and assessed by researchers to organize the next co‐creation stage.

### 3.2 Co‐design (Session Lasting 1 h and 30 min [V2])

The main objective of this stage was to design some of the nutritional and PA recommendations based on the suggestions of the co‐ideation stage.

First, the volunteers designed a 5‐day lunch and dinner menu (3 weekly days and 2 weekend days). The nutritionists provided them with several cards showing different dishes and they had to choose those that best suited their preferences. Table S3 shows the menu designed by the volunteers in the co‐design stage according to their preferences.

Second, the researchers showed the volunteers different nutritional and PA recommendation formats adapted to their preferences so that the volunteers could comment on which design was easiest to follow. The nutritional recommendation proposals were conventional menus, menus with pictures of the dishes, and a list of dishes with pictures, whereas the PA recommendation proposals were exercise videos, exercise videos with explanations and online fitness classes. Third, the volunteers voted to decide which nutritional and PA recommendations proposals were the best. In addition, the volunteers were asked for their opinions about some examples of reminders elaborated by the researchers. The most popular proposals were menus with pictures accompanied by a list of dishes with images and online fitness classes. Regarding the reminders, the volunteers positively valued the examples of reminders that were shown to them.

### 3.3 Co‐Implementation (Intervention Lasting 3 Weeks, Introductory Visit [V3])

The objective of the co‐implementation stage was to test the acceptance of the co‐created recommendations compared to standard recommendations, which had the same requirements but were created by the nutritionists of the research team, over a period of 3 weeks. At this stage, it was evaluated whether the co‐created recommendations were adapted to the real needs of the study volunteers compared to the standard recommendations. The volunteers were randomly allocated into two groups (the co‐created recommendations group and standard recommendations group) by a computerized random number generator (randomizer.org) made by an independent statistician. Before starting the co‐implementation, the volunteers in each group received an explanation of the allocated recommendations.

The final nutritional and PA co‐created recommendations were as follows: advice on how to eat healthily, meal structure, a list of NFOC‐diet foods, two examples of menus with pictures, a list of dishes with images and healthy recipes, general PA recommendations (total minutes per week and examples of session planning), advice on how to be active, advice on how to turn daily activities into physical exercises, a list of sports centres in the city and a list of videos with online fitness classes. The standard recommendations were as follows: meal structure, a list of NFOC‐diet foods and general PA recommendations (total minutes per week and examples of session planning).

During the 3‐week co‐implementation stage, the volunteers who co‐implemented received a reminder in the second week to increase their adherence to the nutritional and PA recommendations. Finally, no problems were detected or reported in relation to the co‐created recommendations by the volunteers during the co‐implementation, so the recommendations seem to have been adapted to the necessities and barriers identified in the co‐ideation stage.

### 3.4 Co‐evaluation (Session Lasting 40 min [V4])

The objective of the co‐evaluation stage was to assess the usability of the recommendations and the volunteers' SEE during the co‐creation process. Additionally, adherence to nutritional and PA recommendations was assessed as an extra analysis to obtain more information about the applicability and feasibility of these recommendations in real life.

In the usability assessment, items 3 (*p* = 0.035), 7 (*p* = 0.014) and 10 (*p* = 0.014) were statistically significantly higher for the volunteers in the co‐ideation/co‐design stages compared to the volunteers in the co‐implementation stage (Table 4). The same results were observed with regard to the volunteers' SEE when participating in the co‐creation process in items 3 (*p* = 0.051), 9 (*p* = 0.035) and 14 (*p* = 0.035) (Table 4). However, all volunteers had high scores for both parameters (Table 4). Furthermore, when evaluating if there were differences between the volunteers in the co‐implementation stage who received either the co‐created recommendations or the standard recommendations, no significant differences were found between groups regarding the usability of the recommendations and the volunteers' SEE (Table S4).

**Table 4:** Assessment of usability of the recommendations and volunteers' SEE.

	Co‐ideation and co‐design volunteers (*n* = 7)	Co‐implementation volunteers (*n* = 3)	*p‐*value*
	Mean (IQR)	Mean (IQR)	
**Usability**			
Item 1	4.86 (4–5)	4.33 (3–5)	0.295
Item 2	4.71 (4–5)	4.17 (3–5)	0.234
Item 3	4.86 (4–5)	4.00 (3–5)	0.035
Item 4	4.29 (3–5)	3.50 (1–5)	0.295
Item 5	4.86 (4–5)	4.33 (3–5)	0.534
Item 6	—	—	—
Item 7	4.57 (4–5)	3.33 (2–4)	0.014
Item 8	4.67 (4–5)	4.17 (3–5)	0.485
Item 9	—	4.00 (3–5)	—
Item 10	4.71 (4–5)	3.67 (3–4)	0.014
**SEE**			
Item 1	6.83 (6–7)	5.67 (4–7)	0.093
Item 2	7.00 (7–7)	6.17 (5–7)	0.138
Item 3	7.00 (7–7)	6.00 (5–7)	0.051
Item 4	6.71 (6–7)	6.17 (4–7)	0.445
Item 5	6.71 (6–7)	6.17 (5–7)	0.234
Item 6	6.71 (6–7)	6.50 (6–7)	0.534
Item 7	7.00 (7–7)	6.83 (6–7)	0.628
Item 8	7.00 (7–7)	—	—
Item 9	6.86 (6–7)	6.00 (5–7)	0.035
Item 10	6.86 (6–7)	6.50 (6–7)	0.295
Item 11	6.86 (6–7)	6.17 (5–7)	0.101
Item 12	6.71 (6–7)	6.00 (5–7)	0.181
Item 13	6.86 (6–7)	6.00 (5–7)	0.101
Item 14	6.71 (6–7)	5.67 (5–7)	0.035
Item 15	6.57 (5–7)	6.67 (6–7)	1.000

Abbreviations: SEE, satisfaction and engagement experience; IQR, interquartile range.

* U the Mann–Whitney *U* test: differences between co‐ideation/co‐design volunteers and co‐implementation volunteers; *p*‐value < 0.05 was statistically significant. For those items that were not answered in both groups or in one of the two groups, the *p*‐value of the difference could not be calculated.

The additional analysis of adherence to the nutritional recommendations' with the FFQ showed no significant differences in the micronutrient content between the beginning and the end of the co‐implementation of each group or between the volunteers that co‐implemented the standard recommendations and the co‐created recommendations. Even so, although there were no significant differences, the content of leucine, polyunsaturated fatty acids, vitamin D, sodium, calcium, magnesium, phosphorus, iron and zinc increased in the group that co‐implemented the co‐created recommendations at the end of this stage compared to the beginning. In contrast, in the group that co‐implemented the standard recommendations, the intake of these micronutrients did not increase (Table S5). Focusing on PA recommendations, no significant differences were shown between the beginning and the end of the co‐implementation stage and also between groups. However, volunteers from the co‐created recommendations group improved their PA level from medium to high at the end of the co‐implementation stage. Conversely, no volunteers from the standard recommendations group increased their PA level (Table S6).

## 4 Discussion

This study is the first instance, to our knowledge, to actively involve volunteers participating in a nutritional intervention study (FOOP‐Sarc project) in the co‐creation of nutritional and PA recommendations to be applied in the project to improve sarcopenia parameters. The active involvement of these volunteers in the co‐creation process, especially those who participated in the co‐ideation and co‐design stages, led to the high usability of the co‐created recommendations and high volunteer SEE. Moreover, the co‐implementation stage demonstrated the feasibility of using co‐created recommendations with this target population. For all these reasons, the volunteers of the FOOP‐Sarc project will use the co‐created recommendations.

The choice of an appropriate participatory research methodology is important in this type of study. Co‐creation differs from other participatory research methodologies because it enables the direct and unlimited participation of volunteers [20]. This allows for the detection of problems, needs and barriers, and for co‐created solutions to be selected, designed, tested and assessed in terms of their usability [11]. Co‐creation also facilitates empowerment and encourages equal collaboration among volunteers during the process, which can be crucial for the acceptance and success of implementation [39]. One scoping review stated that group workshops, discussion meetings, open questions, focus group discussions or multidisciplinary debates could be useful techniques for the first stages of co‐creation such as co‐ideation and co‐design [40]. In the present study, during the co‐ideation and co‐design stages, the volunteers were able to give their opinions to design the recommendations taking into account their nutritional and PA habits. In addition, possible barriers for volunteers and other people in their environment when following the recommendations were discussed. Solutions to avoid barriers were also sought for each case. The researchers also used some co‐creation methodologies such as semi‐structured group interviews and customer journey techniques. Ultimately, the co‐created recommendations were designed according to volunteers' preferences. Thus, all the volunteers considered the recommendations highly usable.

According to a scoping review of co‐creation in health research, negative feelings, like pressure and frustration, experienced by the volunteers who participated in the co‐creation process or a lack of time to successfully implement the co‐created materials or interventions were the most common difficulties reported [40]. In the present study, the SEE was high in all volunteers. However, considering the higher SEE values of the volunteers who co‐ideated and co‐designed the recommendations, the volunteers who only co‐implemented may have felt overwhelmed because of the high amount of information they received. Therefore, the amount of information provided is an important point to consider in future co‐creation processes. Also, volunteers who participated in the co‐ideation and co‐design stages obtained higher scores in SEE compared to volunteers who only participated in the co‐implementation phase. The higher SEE scores may have been because although they did not have to implement the recommendations and did not feel overwhelmed, their opinions were listened to and taken into account.

In addition, adherence to nutritional recommendations is a crucial aspect of nutritional intervention studies, yet adherence decreases over the months [41, 42]. Therefore, it is important to enhance adherence and motivate volunteers in interventions by using methodologies of participatory research such as co‐creation to obtain positive health outcomes by identifying barriers to behavioural changes [43]. In the co‐implementation stage of the present study, it can be observed that the co‐created recommendations were accepted well by the volunteers, and an additional analysis showed that volunteer adherence was higher in the co‐created recommendations group compared to the standard recommendations group.

In this context, a parallel randomized controlled trial in which patients, clinicians and researchers co‐created prostate cancer screening patient education materials obtained similar results to the present study [27]. A reduced sample of volunteers participated in brainstorming and designing the patient education materials, while a different, larger sample of volunteers tested the co‐created recommendations to assess their effectiveness compared to the recommendations created by researchers. However, the effectiveness of the co‐created support materials was similar to the education materials created by the researchers, although the co‐created materials received higher usability scores and the volunteers preferred them compared to the standard materials created by researchers [27]. Another randomized clinical trial showed the feasibility and acceptance of an intervention to reduce sedentary behaviour co‐created by home residents and some stakeholders; the co‐created intervention reduced sedentary behaviour and improved quality of life compared to a control intervention based on usual care [44]. A further co‐creation study used workshops, focus groups and semi‐structured interviews to co‐create an electronic informed consent interface that was personalized to volunteers' needs [45]. The interface enhanced the communication among participants and researchers during and after the clinical study, keeping people updated about the results obtained [45]. Moreover, a systematic review of co‐creation initiatives during the COVID‐19 pandemic highlighted the positive results of implementing this methodology in ambulatory care. The systematic review concluded that co‐creation improved the quality of care, emphasizing the importance of involving patients in healthcare research to conduct patient‐centred interventions as a way of changing ambulatory care [46].

A major strength of the present study is its status as one of the first nutritional interventional studies to use the co‐creation process to achieve higher volunteer engagement and satisfaction while increasing the usability of the co‐created recommendations, which represents a novel contribution to healthcare research. For these reasons, the co‐created recommendations were applied to the FOOP‐Sarc project, in which adherence to the recommendations is analysed in the context of the global sample of the nutritional intervention study and its implication on clinical outcomes such as sarcopenia parameters, anthropometric and vascular parameters, blood pressure, glucose and lipid parameters, inflammation biomarkers, renal function and quality of life. According to the scientific literature, the most effective strategies for tackling sarcopenia are a high‐quality diet and regular PA [22]. Therefore, considering that co‐created nutritional and PA recommendations follow these criteria, their integration into clinical practice could be used for patient‐centred care, reinforcing their adherence to the intervention and enhancing all sarcopenia clinical outcomes (muscle strength, muscle mass and physical performance).

Considering the positive results related to usability and volunteer SEE obtained in the present study, it will be interesting to include participatory research in future nutritional intervention studies, particularly the co‐creation, which involves the active participation of volunteers in the development of programmes, interventions, recommendations, etc. [11, 39]. Furthermore, in the future, it could be useful to include co‐creation in clinical studies to increase the feasibility of recommendations.

The present study had some limitations. First, although the scientific literature suggests that a sample of 10–12 subjects is enough to carry out a co‐creation process [18], when focusing on our statistical analysis, the small sample in the co‐implementation stage (*n* = 6) made it difficult to obtain statistically significant differences between the beginning and the end of the co‐implementation stage or between groups. Thus, the nonsignificant results on adherence are not robust and do not allow for generalization. Second, the fact that different volunteers participated in the co‐ideation/co‐design stages and the co‐implementation stages may have affected the satisfaction of volunteers who co‐designed the recommendations but did not test them. Third, all volunteers who participated in the co‐creation were active people who practiced some level of PA. If we had included some sedentary people or people with different levels of sarcopenia severity, the co‐ideation and co‐design stages could have been different. Fourth, stakeholders such as primary healthcare professionals, geriatricians, physiotherapists or relatives of the elderly participants were not included in the co‐creation process, which could be a potential improvement in future co‐creation processes involving older people. Due to the absence of these stakeholders, it was not possible to use other resources or receive different points of view on the needs and barriers of older people from professionals who deal with them daily. Finally, feelings of overwhelm could be a potential challenge during the co‐creation process and should be addressed in future co‐creation studies. Thus, future studies should address all these limitations by increasing the sample size, employing more robust statistical methods, including sedentary people and people with different levels of sarcopenia severity in the co‐creation process, involving stakeholders related to older people, and using validated questionnaires to strengthen the credibility of the findings.

## 5 Conclusion

As a participatory research approach, the use of co‐creation in a nutritional intervention study demonstrates both the high usability of the co‐created recommendations for sarcopenia and high levels of SEE in volunteers who participated in the co‐creation process, particularly those who participated in the co‐ideation (s1) and co‐design (s2) stages, which are key stages in the co‐creation process. In addition, the co‐created recommendations were well accepted by the volunteers in the co‐implementation stage, demonstrating their feasibility. The present study demonstrates that co‐creation has great utility in creating recommendations to be included in nutritional intervention studies. Incorporating this methodology in clinical study designs would have a great effect on increasing the adherence of volunteers. However, the final results of the FOOP‐Sarc project, taking into account its global sample size, are needed to assess the volunteer adherence and the effectiveness of the co‐created recommendations. Despite this, participatory research should be applied not only in the co‐creation of intervention recommendations but in all phases of research conducted with volunteers.

## Author Contributions

**Maria Besora‐Moreno:** conceptualization, data curation, formal analysis, funding acquisition, investigation, methodology, visualization, writing – original draft. **Cristina Sepúlveda:** formal analysis, investigation, methodology, visualization, writing–original draft. **Judit Queral:** visualization, writing – review and editing. **Claudia Jiménez‐ten Hoevel:** formal analysis, investigation, methodology, visualization, writing–original draft. **Anna Pedret:** conceptualization, data curation, formal analysis, investigation, methodology, supervision, visualization, writing – original draft. **Lucia Tarro:** visualization, writing – review and editing. **Rosa M. Valls:** conceptualization, data curation, formal analysis, funding acquisition, investigation, methodology, project administration, resources, supervision, visualization, writing – original draft. **Rosa Solà:** data curation, conceptualization, formal analysis, funding acquisition, methodology, investigation, project administration, resources, supervision, visualization, writing –original draft. **Elisabet Llauradó:** conceptualization, data curation, formal analysis, investigation, methodology, supervision, visualization, writing – original draft.

## Ethics Statement

The study was approved by the Ethics Committee for Research with Medicines (Comité de Ética de Investigación con medicamentos – CEIm) (033/2020). Also, was in accordance with the Declaration of Helsinki and all volunteers signed the informed consent before participation.

## Conflicts of Interest

The authors declare no conflicts of interest.

## Supporting information

Supporting information.

## Data Availability

The datasets generated and/or analysed during the current study are not publicly available due to data confidentially but are available from the corresponding author on reasonable request.
