# Supplementary material for: Participatory Research in Clinical Studies: An Innovative Approach to Co‐creating Nutritional and Physical Activity Recommendations for Older Adults With Sarcopenia (FOOP‐Sarc Project)
Source: Health Expect. 2025 Apr 4;28(2):e70187. doi: 10.1111/hex.70187 (PMC11970529; doi:10.1111/hex.70187)
Supplement: Supplementary file 1 — Supporting information. [file HEX-28-e70187-s001.docx]

**Participatory research in clinical studies: an innovative approach to co-creating nutritional and physical activity recommendations for older adults with sarcopenia (FOOP-Sarc project)**

| **Supplementary Table 1. Food frequency questionnaire of FOOP-Sarc study** | | | | | | | | | |
| --- | --- | --- | --- | --- | --- | --- | --- | --- | --- |
| In the last two weeks, how often have you eaten the following foods: | | | | | | | | | |
|  | **Never** | **Daily** | | | **Weekly** | | | | |
|  |  | **1** | **2** | **>2** | **1** | **2** | **3** | **4** | **≥5** |
| **MEAT AND BY-PRODUCTS** | | | | | | | | | |
| Chicken, turkey (100-150 g) |  |  |  |  |  |  |  |  |  |
| Rabbit (100-150 g) |  |  |  |  |  |  |  |  |  |
| Pork, beef, lamb (100-150 g) |  |  |  |  |  |  |  |  |  |
| Salt cured ham, ham... (30-50 g) |  |  |  |  |  |  |  |  |  |
| **FISH AND SEAFOOD** | | | | | | | | | |
| Sole, hake, cod, bass, monkfish, halibut, panga, sea bream… (100-150 g) |  |  |  |  |  |  |  |  |  |
| Tuna, salmon, anchovy, mackerel, sardine... (100-150 g) |  |  |  |  |  |  |  |  |  |
| Canned fish: tuna, sardine, anchovy… (1 can) |  |  |  |  |  |  |  |  |  |
| Mussels/clams (medium portion) |  |  |  |  |  |  |  |  |  |
| Squids/cuttlefish (100-150 g) |  |  |  |  |  |  |  |  |  |
| Shrimp/lobster/prawn (100 g) |  |  |  |  |  |  |  |  |  |
| **EGGS** | | | | | | | | | |
| Egg (1 unit) |  |  |  |  |  |  |  |  |  |
| **DAIRY AND BY-PRODUCTS** | | | | | | | | | |
| Whole/semi-skimmed/skimmed milk (200 ml) |  |  |  |  |  |  |  |  |  |
| Whole natural/skimmed yogurt (125 g) |  |  |  |  |  |  |  |  |  |
| Fresh cheese (50-70 g) |  |  |  |  |  |  |  |  |  |
| Goat/cured manchego/semi-cured cheese (50 g) |  |  |  |  |  |  |  |  |  |
| Gouda/edam/Emmental cheese (50 g) |  |  |  |  |  |  |  |  |  |
| **VEGETABLE DRINKS** | | | | | | | | | |
| Soy drink (200 ml) |  |  |  |  |  |  |  |  |  |
| **CEREALS AND BY-PRODUCTS** | | | | | | | | | |
| White bread (60 g) |  |  |  |  |  |  |  |  |  |
| Wholemeal bread (60 g) |  |  |  |  |  |  |  |  |  |
| White rice (80 g raw or medium portion) |  |  |  |  |  |  |  |  |  |
| Wholemeal rice (80 g raw or medium portion) |  |  |  |  |  |  |  |  |  |
| Pasta (80 g raw or medium portion) |  |  |  |  |  |  |  |  |  |
| Wholemeal pasta (80 g raw or medium portion) |  |  |  |  |  |  |  |  |  |
| Quinoa (80 g raw or medium portion) |  |  |  |  |  |  |  |  |  |
| Rusk (30 g) |  |  |  |  |  |  |  |  |  |
| Canned corn (1 individual can) |  |  |  |  |  |  |  |  |  |
| Oat flakes (30-40 g) |  |  |  |  |  |  |  |  |  |
| **TUBERS** | | | | | | | | | |
| Potato/sweet potato (1 medium size) |  |  |  |  |  |  |  |  |  |
| **LEGUMES** | | | | | | | | | |
| Peas (80 g) |  |  |  |  |  |  |  |  |  |
| Kidney beans, chickpeas, lentils (80 g) |  |  |  |  |  |  |  |  |  |
| Soybeans (80g) |  |  |  |  |  |  |  |  |  |
| Lupin (80 g) |  |  |  |  |  |  |  |  |  |
| **NUTS AND SEEDS** | | | | | | | | | |
| Almonds/hazelnuts/walnuts/pine nuts (30 g or a handful) |  |  |  |  |  |  |  |  |  |
| Pistachio (30 g or a handful) |  |  |  |  |  |  |  |  |  |
| Peanut (30 g or a handful) |  |  |  |  |  |  |  |  |  |
| Peanut butter (20 g o 1 tablespoon) |  |  |  |  |  |  |  |  |  |
| Sesame/sunflower/chia/flax seeds (20-30 g) |  |  |  |  |  |  |  |  |  |
| **VEGETABLES AND FRUITS** | | | | | | | | | |
| Swiss chard/spinach (60-80 g or a medium dish) |  |  |  |  |  |  |  |  |  |
| Rocket/[lamb's lettuce](https://www.linguee.es/ingles-espanol/traduccion/lamb%27s+lettuce.html) (60-80 g or a medium plate) |  |  |  |  |  |  |  |  |  |
| Mushrooms (140-150 g as a side dish) |  |  |  |  |  |  |  |  |  |
| Tomato (100-150 g or medium size) |  |  |  |  |  |  |  |  |  |
| Banana (150-200g) |  |  |  |  |  |  |  |  |  |
| Avocado (1 medium unit; 150-200 g) |  |  |  |  |  |  |  |  |  |
| **FORTIFIED FOODS** | | | | | | | | | |
| Fortified milk (200 ml) |  |  |  |  |  |  |  |  |  |
| Fortified cereals (30-40 g) |  |  |  |  |  |  |  |  |  |
| **OTHERS** | | | | | | | | | |
| Tofu (150 g) |  |  |  |  |  |  |  |  |  |
| Tempeh (150 g) |  |  |  |  |  |  |  |  |  |
| Pure cocoa (15-20 g, 2 ground tablespoons or 1 large tablespoon) |  |  |  |  |  |  |  |  |  |
| Date (2-3 units) |  |  |  |  |  |  |  |  |  |
| Raisins (40g or a handful) |  |  |  |  |  |  |  |  |  |

| **Supplementary Table 2. Customer journey** | | | | | | | | | | | | | | | | | | | | | | |  |
| --- | --- | --- | --- | --- | --- | --- | --- | --- | --- | --- | --- | --- | --- | --- | --- | --- | --- | --- | --- | --- | --- | --- | --- |
| 1. Mark with a cross the box according to the time of day when you do the following meals or activities: | | | | | | | | | | | | | | | | | | | | | | |  |
| **Hours** | | **6 h** | **7 h** | **8 h** | **9 h** | **10 h** | **11 h** | | **12 h** | **13 h** | **14 h** | **15 h** | **16 h** | **17 h** | **18 h** | | **19 h** | **20 h** | **21 h** | **22 h** | **23 h** | **24 h** |  |
| **Meal distribution** | |  |  |  |  |  |  | |  |  |  |  |  |  |  | |  |  |  |  |  |  |  |
| Breakfast | |  |  |  |  |  |  | |  |  |  |  |  |  |  | |  |  |  |  |  |  |  |
| Mid-morning | |  |  |  |  |  |  | |  |  |  |  |  |  |  | |  |  |  |  |  |  |  |
| Lunch | |  |  |  |  |  |  | |  |  |  |  |  |  |  | |  |  |  |  |  |  |  |
| Afternoon snack | |  |  |  |  |  |  | |  |  |  |  |  |  |  | |  |  |  |  |  |  |  |
| Dinner | |  |  |  |  |  |  | |  |  |  |  |  |  |  | |  |  |  |  |  |  |  |
| Others (¿Which?...............................................) | |  |  |  |  |  |  | |  |  |  |  |  |  |  | |  |  |  |  |  |  |  |
| **Food groups** | |  |  |  |  |  |  | |  |  |  |  |  |  |  | |  |  |  |  |  |  |  |
| Meat and by-products (including cold meat) | |  |  |  |  |  |  | |  |  |  |  |  |  |  | |  |  |  |  |  |  |  |
| Fish and by-products | |  |  |  |  |  |  | |  |  |  |  |  |  |  | |  |  |  |  |  |  |  |
| Egg | |  |  |  |  |  |  | |  |  |  |  |  |  |  | |  |  |  |  |  |  |  |
| Dairy products and by-products | |  |  |  |  |  |  | |  |  |  |  |  |  |  | |  |  |  |  |  |  |  |
| Cereals and by-products | |  |  |  |  |  |  | |  |  |  |  |  |  |  | |  |  |  |  |  |  |  |
| Legumes | |  |  |  |  |  |  | |  |  |  |  |  |  |  | |  |  |  |  |  |  |  |
| Nuts and by-products, and seeds | |  |  |  |  |  |  | |  |  |  |  |  |  |  | |  |  |  |  |  |  |  |
| Fortified foods (milk, cereals, etc.) | |  |  |  |  |  |  | |  |  |  |  |  |  |  | |  |  |  |  |  |  |  |
| Others (tofu, tempeh, etc.) (¿Which?...............) | |  |  |  |  |  |  | |  |  |  |  |  |  |  | |  |  |  |  |  |  |  |
| **Type of physical activity** | |  |  |  |  |  |  | |  |  |  |  |  |  |  | |  |  |  |  |  |  |  |
| Stretched, flexibility exercises | |  |  |  |  |  |  | |  |  |  |  |  |  |  | |  |  |  |  |  |  |  |
| Walk | |  |  |  |  |  |  | |  |  |  |  |  |  |  | |  |  |  |  |  |  |  |
| Hiking | |  |  |  |  |  |  | |  |  |  |  |  |  |  | |  |  |  |  |  |  |  |
| Running or jogging | |  |  |  |  |  |  | |  |  |  |  |  |  |  | |  |  |  |  |  |  |  |
| Exercises with weights | |  |  |  |  |  |  | |  |  |  |  |  |  |  | |  |  |  |  |  |  |  |
| Swimming | |  |  |  |  |  |  | |  |  |  |  |  |  |  | |  |  |  |  |  |  |  |
| Aquagym | |  |  |  |  |  |  | |  |  |  |  |  |  |  | |  |  |  |  |  |  |  |
| Bike | |  |  |  |  |  |  | |  |  |  |  |  |  |  | |  |  |  |  |  |  |  |
| Dance | |  |  |  |  |  |  | |  |  |  |  |  |  |  | |  |  |  |  |  |  |  |
| Tai-Chi or yoga | |  |  |  |  |  |  | |  |  |  |  |  |  |  | |  |  |  |  |  |  |  |
| Others (¿Which?............................................) | |  |  |  |  |  |  | |  |  |  |  |  |  |  | |  |  |  |  |  |  |  |
| **Sedentary time** | |  |  |  |  |  |  | |  |  |  |  |  |  |  | |  |  |  |  |  |  |  |
| Reading | |  |  |  |  |  |  | |  |  |  |  |  |  |  | |  |  |  |  |  |  |  |
| With the computer | |  |  |  |  |  |  | |  |  |  |  |  |  |  | |  |  |  |  |  |  |  |
| Watching TV | |  |  |  |  |  |  | |  |  |  |  |  |  |  | |  |  |  |  |  |  |  |
| With the mobile | |  |  |  |  |  |  | |  |  |  |  |  |  |  | |  |  |  |  |  |  |  |
| Others (¿Which?...............................................) | |  |  |  |  |  |  | |  |  |  |  |  |  |  | |  |  |  |  |  |  |  |
| 1. Complete or check the appropriate box: | | | | | | | | | | | | | | | | | | | | | | | |
| **Meal distribution** | **Food groups** | | | | | | | **Physical activity** | | | | | | | | **Sedentary time** | | | | | | | |
| **I eat an average of ____ times a day** | **My diet is rich in…**  □ meat and by-products  □ fish and by-products  □ eggs  □ dairy products and by-products  □ cereals and by-products  □ legumes  □ nuts and seeds | | | | | | | **I do an average of _____ minutes/hours a day of physical activity** | | | | | | | | **I am sitting an average of _____ minutes/hours a day** | | | | | | | |
| **During the weekend I make the following meals…**  □ breakfast  □ mid-morning  □ lunch  □ afternoon snack  □ dinner | **I usually eat…**  □ wholemeal pasta/rice  □ wholemeal bread  □ none | | | | | | | **I do physical activity in…**  □ house  □ the park  □ a gym or sports club  □ a civic center  □ the street  □ other___________________ | | | | | | | | **I am more time sitting….**  □ during the week  □ on weekends | | | | | | | |
| **If I miss a meal, it’s usually…**  □ breakfast  □ mid-morning  □ lunch  □ afternoon snack  □ dinner  □ none |  |  |  |  |  |  |  | **I do more physical activity…**  □ during the week  □ on weekends  □ none | | | | | | | | **I spend the most time sitting…**  □ alone  □ with relatives  □ with friends | | | | | | | |
| **I could add a new food or dish in…**  □ breakfast  □ mid-morning  □ lunch  □ afternoon snack  □ dinner  □ none |  | | | | | | | **I do more physical activity…**  □ alone  □ with relatives  □ with friends  □ none | | | | | | | |  |  |  |  |  |  |  |  |

| **Supplementary Table 3. Menu designed by the volunteers in the co-design stage** | | | | | |
| --- | --- | --- | --- | --- | --- |
|  | | | | | |
|  | **Weekly day** | **Weekly day** | **Weekly day** | **Weekend day** | **Weekend day** |
| **Lunch** | - Chicken with mashed potatoes - Meat cannelloni with bechamel sauce - Turkey meatballs with vegetables - Cream of mushroom soup - Rice with lentils and pistachios - Chard with potato - Spaghetti carbonara | - Beef steak with Brussels sprouts - Rabbit with tomato sauce - Spinach cannelloni - Spaghetti with zucchini, mushrooms and carrot - Chicken with almond sauce - Spaghetti *“a la marinera”* - Stewed rabbit with onion | - Stew with potatoes and vegetables - Chicken curry - Baked potatoes with cod - Spaghetti with pistachio pesto - Lupine salad with cucumber and olives | - Broccoli with cured ham and pine nuts - Baked chicken with potatoes au gratin with four cheeses - Broccoli, walnut and cheese lasagna - Cheeks with poma compote - Rabbit in curry sauce | - Macaroni Bolognese - Spaghetti with asparagus and walnuts - Arugula, pear and cheese salad - Grilled lamb with asparagus - Cuttlefish with artichokes - Rice with textured soy - Tuna and tomato cannelloni |
| **Dinner** | - Sardines with white beans and grilled tomato - Guacamole with carrot and cucumber crudités - Hake in green sauce - Arugula, pear, pistachio and cheese salad - Lettuce, sweet corn, tomato and onion salad | - Caesar salad with tofu - Salad with goat cheese and walnuts - Pasta with chicken and spinach - Turkey skewers with basmati rice - Quinoa salad with spinach and hazelnuts | - Potato, tuna and hard-boiled egg salad - Mushroom and vegetable skewers - Turkey stuffed with ham and cheese - Rice salad with peas, carrots and sweet corn | - Eggs stuffed with tuna and shrimp - Green bean cream with sunflower seeds and pistachios - Mushroom and walnut pate - Arugula, apple and raisin salad - Lamb's lettuce, cheese and walnut sandwich | - Mushroom and hazelnut cream - Toast with cheese and avocado - Salmon with spinach |

| **Supplementary Table 4. Assessment of usability of the recommendations and volunteers’ SEE from the co-implementation stage.** | | | |
| --- | --- | --- | --- |
|  | Co-created recommendations group (n = 3) | Standard recommendations group (n = 3) | *p-value** |
|  | Mean (IQR) | Mean (IQR) |  |
| **Usability** |  |  |  |
| Item 1 | 4.33 (4-5) | 4.33 (3-5) | 1.000 |
| Item 2 | 3.67 (3-4) | 4.67 (4-5) | 0.200 |
| Item 3 | 3.67 (3-4) | 4.33 (4-5) | 0.400 |
| Item 4 | 4.00 (3-6) | 3.00 (1-4) | 0.700 |
| Item 5 | 3.67 (3-5) | 5.00 (5-5) | 0.200 |
| Item 6 | - | - | - |
| Item 7 | 3.00 (2-4) | 3.67 (3-4) | 0.400 |
| Item 8 | 3.33 (3-4) | 5.00 (5-5) | 0.100 |
| Item 9 | 3.67 (3-4 | 4.33 (4-5) | 0.400 |
| Item 10 | 3.33 (3-4) | 4.00 (4-4) | 0.200 |
| **SEE** |  |  |  |
| Item 1 | 6.00 (5-7) | 5.33 (4-7) | 0.700 |
| Item 2 | 6.00 (5-7) | 6.33 (5-7) | 0.700 |
| Item 3 | 6.00 (5-7) | 6.00 (5-7) | 1.000 |
| Item 4 | 5.67 (4-7) | 6.67 (6-7) | 0.400 |
| Item 5 | 6.33 (6-7) | 6.00 (5-7) | 0.700 |
| Item 6 | 6.33 (6-7) | 6.67 (6-7) | 0.700 |
| Item 7 | 6.67 (6-7) | 7.00 (7-7) | 0.700 |
| Item 8 | - | - | - |
| Item 9 | 6.00 (6-6) | 6.00 (5-7) | 1.000 |
| Item 10 | 6.33 (6-7) | 6.67 (6-7) | 0.700 |
| Item 11 | 6.33 (6-7) | 6.00 (5-7) | 0.700 |
| Item 12 | 6.00 (5-7) | 6.00 (5-7) | 1.000 |
| Item 13 | 6.00 (5-7) | 6.00 (5-7) | 1.000 |
| Item 14 | 6.00 (5-7) | 5.33 (5-6) | 0.400 |
| Item 15 | 6.33 (6-7) | 7.00 (7-7) | 0.200 |
| SEE: satisfaction and engagement experience; IQR: interquartile range.  *U the Mann Whitney: differences between co-ideation/co-design volunteers and co-implementation volunteers; p-value <0.05 was statistically significant. For those items that were not answered in both groups or in one of the two groups, the p-value of the difference could not be calculated. | | | |

| **Supplementary Table 5. Nutritional recommendations adherence at co-implementation stage.** | | | | | | | |
| --- | --- | --- | --- | --- | --- | --- | --- |
|  | Co-created recommendations group (n = 3) | | | Standard recommendations group (n = 3) | | |  |
|  | Beginning  Median (IQR) | End  Median (IQR) | *p-value** | Beginning  Median (IQR) | End  Median (IQR) | *p-value** | *p-value*** |
| Leucine (mg) | 6477.34 (5182.34-7925.25) | 8336.49 (5181.20-13135.80) | 0.285 | 7192.5 (6193.66-8616.87) | 7072.54 (6584.37-8196.31) | 1 | 0.4 |
| PUFAS (g) | 8.88 (5.39-19.99) | 20.95 (5.20-28.54) | 0.285 | 22.14 (11.19-119.99) | 18.81 (13.71-27.48) | 0.285 | 0.2 |
| Vit. D (µg) | 4.26 (1.54-5.99) | 6.69 (1.09-17.07) | 0.285 | 3.58 (2.89-5.74) | 3.3 (3.05-5.34) | 0.285 | 1 |
| Na (mg) | 1323.15 (1213.57-2263.33) | 2790.12 (786.61-3876.33) | 0.285 | 1829.33 (1415.26-2419.77) | 1604.54 (1460.0-2293.46) | 1 | 0.4 |
| K (mg) | 3688.27 (2393.12-4031.17) | 3607.48 (2381.09-6399.66) | 1 | 3874.13 (3207.33-4163.41) | 3120.78 (2819.41-4641.30) | 0.593 | 0.4 |
| Ca (mg) | 916.01 (661.31-1066.64) | 1021.99 (523.71-1778.78) | 0.593 | 1148.74 (1093.02-1282.16) | 1253.26 (830.27-1437.66) | 1 | 0.7 |
| Mg (mg) | 383.29 (231.81-467.63) | 460.02 (223.49-908.28) | 0.285 | 430.21 (420.76-583.26) | 478.67 (335.24-671.90) | 1 | 0.7 |
| P (mg) | 1447.72 (1012.74-1764.98) | 1695.04 (999.51-2833.55) | 0.285 | 1938.61 (1618.35-1988.38) | 1849.38 (1392.55-2317.69) | 1 | 0.7 |
| Fe (mg) | 14.24 (9.04-16.01) | 17.73 (11.27-31.03) | 0.109 | 17.12 (11.42-21.82) | 13.67 (11.95-26.99) | 0.593 | 0.4 |
| Zn (mg) | 10.56 (7.55-11.61) | 12.67 (8.01-22.02) | 0.109 | 13.39 (11.57-13.89) | 12.83 (10.45-16.12) | 1 | 0.7 |
| This table show the information about micronutrient content in the co-designed and standard recommendations group, the difference between beginning and end of the co-implementation stage and the difference among groups.  IQR: interquartile range; PUFAS: polyunsaturated fatty acids; Vit. D: vitamin D; Na: sodium; Ca: calcium; Mg: magnesium; K: phosphorus; Fe: iron; Zn: zinc  *Wilcoxon: differences between beginning and end of the co-implementation stage; **U the Mann Whitney: differences between the standard recommendations group and co-designed recommendations group; p-value <0.05 was statistically significant. | | | | | | | |

| **Supplementary Table 6. Physical activity recommendations adherence at co-implementation stage from the IPAQ-E questionnaire.** | | | | | | |
| --- | --- | --- | --- | --- | --- | --- |
|  | Co-created recommendations group (n = 3) | | | Standard recommendations group (n=3) | | |
| Physical activity*¹* | Beginning n (%) | End  n (%) | *p-value** | Beginning n (%) | End  n (%) | *p-value** |
| 0 | 0 | 0 | 0.25 | 0 | 0 | 0.25 |
| 1 | 3 (100) | 0 |  | 2 (66.7) | 3 (100) |  |
| 2 | 0 | 3 (100) |  | 1 (33.3) | 0 |  |
| This table show the information about physical activity level in the co-designed and standard recommendations group and the difference between beginning and end of the co-implementation stage.  ¹ Physical activity: low (0), medium (1), or high (2)  *Mc Nemar: differences between beginning and end of the co-implementation stage; p-value <0.05 was statistically significant. | | | | | | |
